# A self-perpetuating repressive state of a viral replication protein blocks superinfection by the same virus

**DOI:** 10.1371/journal.ppat.1006253

**Published:** 2017-03-07

**Authors:** Xiao-Feng Zhang, Rong Sun, Qin Guo, Shaoyan Zhang, Tea Meulia, Randal Halfmann, Dawei Li, Feng Qu

**Affiliations:** 1 Department of Plant Pathology, The Ohio State University, Wooster, Ohio, United States of America; 2 State Key Laboratory of Agro-Biotechnology, College of Biological Sciences, China Agricultural University, Beijing, China; 3 Molecular and Cellular Imaging Center, Ohio Agricultural Research and Development Center, The Ohio State University, Wooster, Ohio, United States of America; 4 Stowers Institute for Medical Research, Kansas City, Missouri, United States of America; Agriculture and Agri-Food Canada, CANADA

## Abstract

Diverse animal and plant viruses block the re-infection of host cells by the same or highly similar viruses through superinfection exclusion (SIE), a widely observed, yet poorly understood phenomenon. Here we demonstrate that SIE of turnip crinkle virus (TCV) is exclusively determined by p28, one of the two replication proteins encoded by this virus. p28 expressed from a TCV replicon exerts strong SIE to a different TCV replicon. Transiently expressed p28, delivered simultaneously with, or ahead of, a TCV replicon, largely recapitulates this repressive activity. Interestingly, p28-mediated SIE is dramatically enhanced by C-terminally fused epitope tags or fluorescent proteins, but weakened by N-terminal modifications, and it inversely correlates with the ability of p28 to complement the replication of a p28-defective TCV replicon. Strikingly, p28 in SIE-positive cells forms large, mobile punctate inclusions that trans-aggregate a non-coalescing, SIE-defective, yet replication-competent p28 mutant. These results support a model postulating that TCV SIE is caused by the formation of multimeric p28 complexes capable of intercepting fresh p28 monomers translated from superinfector genomes, thereby abolishing superinfector replication. This model could prove to be applicable to other RNA viruses, and offer novel targets for antiviral therapy.

## Author summary

Superinfection exclusion (SIE) is employed by many viruses to guard their host cells against secondary invasions by the same or highly similar viruses. We describe a transformative discovery that self-perpetuating coalescence of a virus-encoded replication protein serves as a novel mechanism for SIE. Our findings further suggest that the primary targets of SIE may very well be progeny viral genomes rather than superinfectors, with the goal of blocking renewed multiplication of progeny viruses in the cells resided by “parental” viruses, thereby minimizing the proliferation of replication errors. Consequently, the general mechanistic framework revealed in this study may be highly conserved among viruses with diverse genome structures and host organisms, and could become a potent target for antiviral therapy.

## Introduction

Superinfection exclusion (SIE) refers to the ability of a pre-existing virus (the primary invader) to exclude secondary infections by the same or closely related viruses (superinfectors) at cellular and/or organismal levels. SIE has been observed with many human and animal pathogenic viruses, including the reverse-transcribing human immunodeficiency virus (HIV), positive sense (+) RNA viruses such as hepatitis C virus (HCV) and West Nile virus (WNV), as well as negative sense (-) RNA viruses like vesicular stomatitis virus (VSV) [1–5]. More recent studies reported examples of SIE occurring during infections of large, double-stranded DNA viruses including herpesviruses and poxviruses [6, 7]. In all these examples SIE exerted by primary invaders acted in single cells to prevent the multiplication of highly homologous superinfectors in the same cells.

SIE has also been documented for many plant viruses including citrus tristeza virus (CTV) and tobacco mosaic virus (TMV) [8, 9]. Studies using mutants of CTV, plum pox virus, soilborne wheat mosaic virus, and apple latent spherical virus that were labelled with different fluorescent proteins, established that co-introduced variants of the same virus occupy adjacent, yet non-overlapping cell clusters in the same leaves or tissue niches [10–13]. These studies clearly demonstrated that SIE among closely related plant virus variants likewise exclude each other at the cellular level, thus drawing strong parallels between SIEs occurring in plant and animal virus infections.

SIE may also be mechanistically related to the well-documented cross protection phenomenon observed in virus-infected plants [14–16]. Cross protection refers to the protection gained by plants against a more damaging virus variant, through pre-inoculating these plants with a mild variant of the same virus. Although cross protection has been adopted for plant virus disease management for at least 50 years [15], exactly how the protection is achieved remains to be satisfactorily explained. In theory the cellular level SIE could account for at least some aspects of cross protection, as the plant-wide spread of the pre-inoculated variants could block the cells already occupied by these variants from being invaded by the more severe strains [17]. Nevertheless, there is some evidence to suggest that additional mechanisms might act at the organismal level to augment cross protection [10].

This widespread functional conservation of the cellular level SIE suggests that, once better understood, it could become a promising target for antiviral therapy. However, the molecular mechanisms of SIE remain little elucidated for most viruses. While for some viruses interference with entry of the superinfector appears to be critical [18], for most others the essential inhibitory step is clearly post-entry [2, 3, 19]. A striking feature of SIE shared by the viruses examined so far is its dependence on one or a few virus-encoded proteins and relatively little involvement of host cell components, highlighting SIE as a primarily virus-driven phenomenon [6, 20].

**Fig 1:**
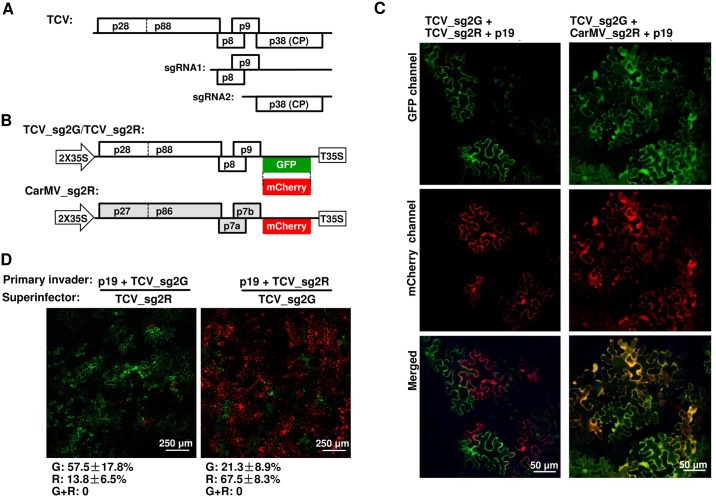
Two TCV-derived replicons exclude each other at the cellular level. See S1 Fig for additional information. (A) Schematic depiction of TCV genome structure. Note that the three 3’ proximal open reading frames (ORFs) encoding p8, p9, and p38 are translated from two subgenomic (sg) RNAs (sgRNA1 and 2). (B) Diagrams of TCV and CarMV replicon constructs expressing GFP and mCherry, used experiments depicted in (C) and (D). (C) Mutual exclusion occurs between TCV_sg2G and TCV_sg2R but not between TCV_sg2G and CarMV_sg2R. Note that a p19-expressing construct was included in this and subsequent experiments to protect the transcribed RNAs from RNA silencing-mediated degradation. (D) Sequential delivery (16-hour interval) allows primary replicons to exclude the superinfecting ones in most cells. See S1D Fig for additional controls. Numbers under the panels represent the percentages of cells fluorescing GFP (G), mCherry (R), or both (G+R) averaged from estimates of at least four different viewing fields. The ranges are standard deviations (SD).

We report here the characterization of molecular mechanism of SIE in turnip crinkle virus (TCV) infected cells. TCV is a small icosahedral plant virus with a (+)-strand RNA genome that encodes five proteins. The 5’ proximal p28 and its C-terminally extended derivative—p88—are both required for viral genome replication (Fig 1A). The two small proteins encoded in the middle of the TCV genome, p8 and p9, are translated from a subgenomic RNA (sgRNA1) and function as cell-to-cell movement proteins (MPs; Fig 1A). Finally, the 3’ proximal p38 is translated from a second sgRNA (sgRNA2) and serves both as the capsid protein (CP) and the TCV-encoded suppressor of RNA silencing (Fig 1A).

TCV p28 is known to play an indispensable role in viral RNA replication, presumably by rearranging mitochondrial outer membrane into partially enclosed viral replication complexes (VRCs) that house itself, p88, viral RNA, as well as cellular factors required for optimal genome amplification [21–24]. In the current study, we uncover a novel role of p28 by showing that it exclusively determines SIE among TCV variants. Strikingly, it does so by forming multimeric intracellular inclusions that inactivate p28 translated from superinfectors, thereby denying superinfectors the chance to replicate. This novel, simple mechanism of SIE could prove to be applicable to many other viruses, and predicts a novel target for antiviral therapy.

## Results

### TCV replicons expressing different fluorescent markers display mutual SIE

To adopt TCV as a model for investigating SIE, we first tagged TCV replicons with two different fluorescent proteins (Fig 1B). Previous studies showed that deletions within the CP coding region of TCV, while compromising viral cell-to-cell and systemic movement, did not affect its replication in single cells [25, 26]. We hence created two TCV replicons, TCV_sg2G and TCV_sg2R, by replacing the 5’ two thirds of the CP coding region with that of GFP and mCherry (Fig 1B). The replicon cDNAs were flanked by the duplicated 35S promoter and terminator (2X35S and T35S) of cauliflower mosaic virus, and inserted into a binary plasmid destined for *Agrobacterium tumefaciens* (pPZP212) [27]. The resulting recombinant plasmids were transformed into *A*. *tumefaciens* strain C58C1 in order to initiate TCV replication in *Nicotiana benthamiana* leaf cells via agro-infiltration. Note that expression of GFP and mCherry proteins from these constructs strictly depends on TCV replication, as the sgRNA2 transcript is only produced during replication (hence the “_sg2” designation in the names of replicon constructs). A carnation mottle virus (CarMV)-derived replicon named as CarMV_sg2R was used as a non-TCV control (Fig 1B), as CarMV and TCV share similar genome organizations yet limited pair-wise sequence identities of encoded proteins (approximately 50%) [28]. Finally, a construct that facilitates non-replicative expression of p19, the tomato bushy stunt virus-encoded suppressor of RNA silencing, was included to counteract RNA silencing-mediated degradation of the primary, 2X35S-driven replicon RNAs [27, 28].

*N*. *benthamiana* leaves infiltrated with the replicon constructs were then inspected by confocal fluorescence microscopy at four days post infiltration (4 dpi). As shown in Fig 1C, leaf patches co-infiltrated with the two TCV replicons (left panels) contained cells that expressed either GFP or mCherry, but were completely devoid of cells that expressed both. Indeed, among thousands of cells inspected in multiple repeat experiments, less than 0.1% of the fluorescent cells fluoresced both green and red. In contrast, approximately 80% of the fluorescent cells observed following co-infiltration of TCV_sg2G and CarMV_sg2R expressed both GFP and mCherry (Fig 1C, right panels). These data illustrate that (i) agro-infiltration efficiently and simultaneously introduced multiple viral constructs into the same leaf cells; and (ii) intracellular, mutual exclusion occurred readily between variants of the same virus (TCV), but not between two distantly related viruses (TCV and CarMV).

Was the mutual exclusion between two TCV replicons caused by SIE, which by definition depends on a temporal lag between the entry of primary invader and superinfector? We speculated that co-introduced replicons might experience varying lengths of post-entry delays before they could initiate replication, thus allowing the ones that replicate first to exert SIE against others in the same cells. To test this idea, we compared the timing of fluorescence emergence in cells treated with 2X35S-GFP, a construct that expresses GFP independent of virus replication, and ΔMP_sg2R, a TCV replicon that expresses mCherry only when viral replication occurs (S1 Fig, panel A in Supporting Information). ΔMP_sg2R differs from TCV_sg2R by harboring a 92 nucleotide (nt) deletion within the MP region, thus restricting its replication to primary infected cells (S1 Fig, panel A). As shown in S1B Fig, replication-independent GFP fluorescence emerged in a few cells at 24 hours post infiltration (hpi), but quickly filled more than 70% of cells by 36 hpi. By contrast, replication-dependent mCherry expression first occurred in a few isolated cells at 48 hpi (white arrow in top right panel), and expanded only gradually thereafter, so that approximately 10%, 23%, and 33% of the cells became red fluorescent at 72, 96, and 120 hpi, respectively (S1 Fig, panel B). To summarize, replication-independent expression of GFP occurred early and synchronously in most cells, whereas replication-dependent expression of mCherry was seriously delayed, and then commenced stochastically in a limited number of cells, over an extended time span.

These observations were confirmed with Western blotting (WB) of leaves treated with the GFP and ΔMP_sg2R constructs separately, using an antibody that reacts with both fluorescent proteins (S1 Fig, panel C). Together these results indicate that replication initiation by a TCV replicon in host cells is delayed relative to replication-independent expression, and the length of delay varied dramatically from cell to cell. While the reason for delays will become evident later in this report, their varying lengths likely permitted one of the co-introduced replicons to commence replication earlier than others in the same cell, thus creating the time lag required for SIE.

We sought to further confirm SIE as the underlying reason for the mutual exclusion using a sequential delivery procedure. As shown in Fig 1D, pre-introduction of either TCV_sg2G or TCV_sg2R dramatically reduced the number of cells that replicated the reciprocal superinfector delivered with a 16-hour delay. As demonstrated in S1 Fig, panel D, delayed introduction did not in itself reduce the chance to initiate replication by replicons, as both replicons introduced with a 16-hour delay relative to the p19 construct initiated replication in a similar number of non-overlapping cells. Together, we conclude that SIE likely accounts for the inability of simultaneously introduced TCV_sg2G and TCV_sg2R to co-replicate in the same cells (Fig 1C).

### TCV p28 alone is responsible for SIE against a co-delivered TCV replicon

**Fig 2:**
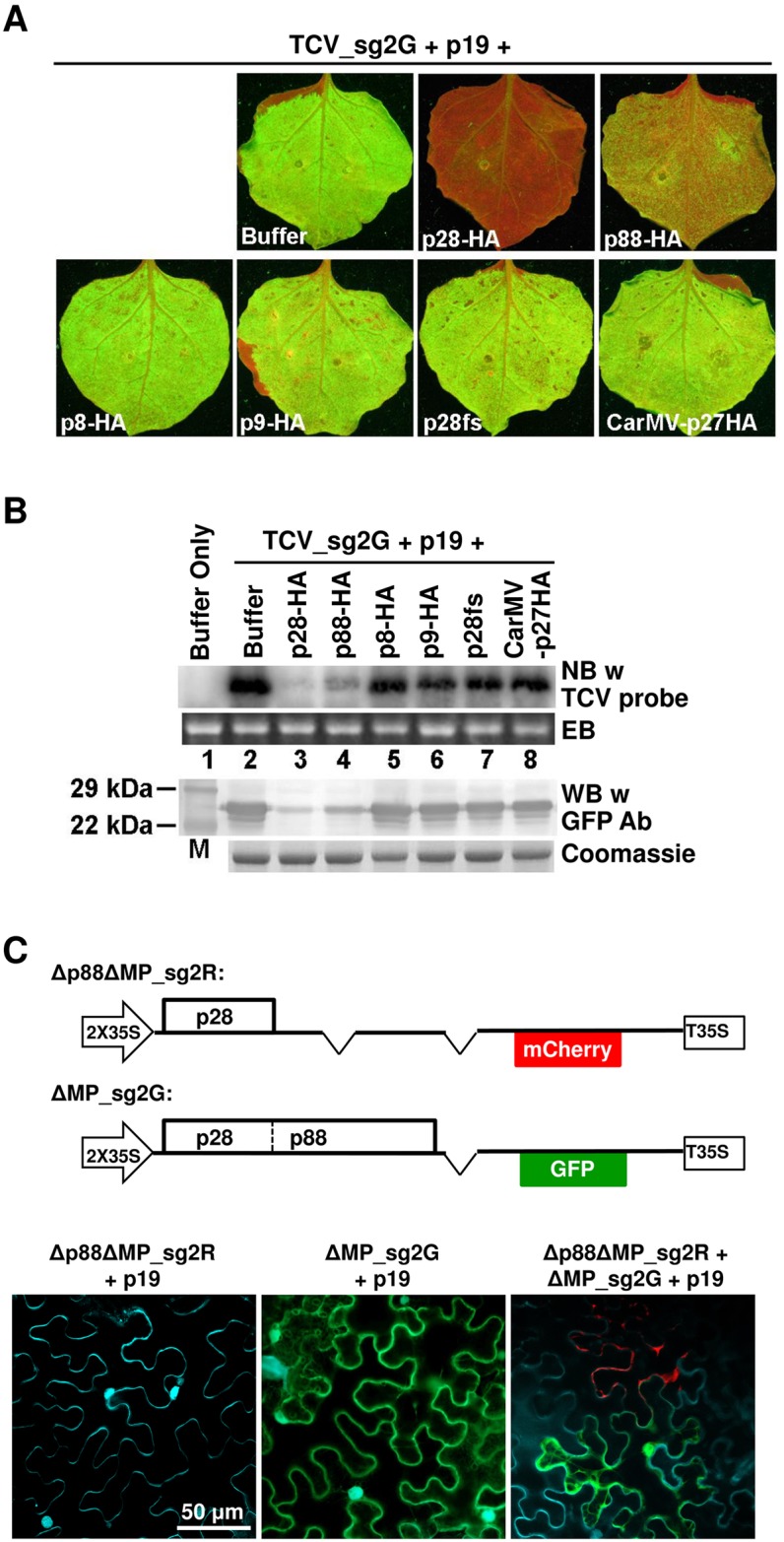
TCV-encoded p28 alone is sufficient to cause the repression of co-introduced TCV replicons. See S2 Fig for additional information. (A) Transiently expressed p28 protein exerts a potent repression on co-introduced TCV_sg2G replicon. Agro-infiltrated leaves were photographed under UV illumination to visualize GFP fluorescence. Names of various constructs tested are shown on respectively panels. Note that all transiently expressed proteins except p19 contain a C-terminal double HA tag. (B) Verification of results in (A) with Northern and Western blottings (NB and WB). EB: ethidium bromide staining of the RNA gel; Coomassie: Coomassie blue staining of the protein gel. (C) Δp88ΔMP_sg2R, a TCV replicon encoding p28 as the sole viral protein, once replicationally rescued by another replicon (ΔMP_sg2G), in turn represses the replication of the latter. Cells were stained with DAPI to show nuclei as well as cell boundaries. Only merged images were shown. See S2B Fig for single channel images of the bottom right panel.

We next assessed whether any of the TCV-encoded proteins could induce SIE by transiently expressing each of the TCV-encoded proteins, along with the TCV_sg2G replicon, in *N*. *benthamiana* cells. TCV p38 (CP) was not included in this set of testing as constructs without CP (TCV_sg2G and TCV_sg2R) still displayed SIE (Fig 1). To facilitate the verification of their expression, these TCV-encoded proteins were all fused to a C-terminal 2XHA tag, permitting WB detection with an anti-HA antibody (S2 Fig, panel A). Expression of p8-HA or p9-HA did not affect TCV_sg2G replication, as evidenced by robust GFP fluorescence in whole leaves (Fig 2A). In contrast, expression of p28-HA eliminated GFP fluorescence. We further established that p28-mediated repression of TCV_sg2G replication depended on the p28 protein, because a frame-shift mutation of p28 (p28fs) abolished repression. Importantly, the repression of TCV_sg2G by p28-HA was highly specific as p27 of CarMV (CarMV-p27HA) was ineffective. Interestingly, p88-HA partially repressed TCV_sg2G accumulation, presumably attributable to its N-terminal region identical to p28 (Fig 1A). We confirmed these results with Northern blots (NB) and WB (Fig 2B). Both TCV_sg2G RNA and GFP protein accumulated to high levels in leaves co-infiltrated with p8-HA, p9-HA, p28fs, or CarMV-p27HA constructs (lanes 5–8), but to much lower levels in leaves expressing p88-HA (lane 4), and were almost undetectable in samples expressing p28-HA (lane 3). Together these data indicate that p28-HA suffices for highly specific, potent repression of TCV replication.

To further test if p28 produced during TCV replication likewise represses the replication of a co-delivered replicon, we generated Δp88ΔMP_sg2R, a derivative of ΔMP_sg2R that contained a 4-nt deletion in the p88 ORF (Fig 2C). As a result, this defective replicon encodes p28 as the sole TCV protein, whose replication function was previously shown to depend on its expression in *cis* [22]. Functional p88 protein must then be supplied through the co-delivered ΔMP_sg2G (Fig 2C). We wish to emphasize that this latter replicon can replicate by itself as it encodes both wild-type p28 and p88. Also keep in mind that the ΔMP deletion in both constructs restricts them to single cells, thus successful complementation must depend on the co-entry of both constructs into the same cells. As expected, cells infiltrated with Δp88ΔMP_sg2R alone did not express mCherry, confirming its inability to replicate (Fig 2C, bottom left). Conversely, cells infiltrated with ΔMP_sg2G alone expressed only GFP (Fig 2C, middle panel). However, when the two constructs were co-infiltrated into the same cells, approximately 10% of fluorescent cells expressed mCherry, indicating that in these cells the replication of Δp88ΔMP_sg2R was successfully rescued *in trans* by the p88 protein translated from ΔMP_sg2G transcripts (Fig 2C, bottom right; see S2 Fig, panel B for images of individual channels). Strikingly, these mCherry-expressing cells always lacked GFP fluorescence, indicating that the replication of ΔMP_sg2G was abolished in these cells (S2 Fig, panel B). This result was consistently observed in multiple repeat experiments, and indicates that Δp88ΔMP_sg2R, once becoming replicationally active, turned around to block the replication of ΔMP_sg2G in the same cells, even though it must rely on the p88 provided by the latter for replication. Together these data reveal two vital insights into TCV SIE: (i) p28 acts both as a replication facilitator for Δp88ΔMP_sg2R itself, and as a repressor in *trans* for ΔMP_sg2G in the same cell, (ii) p28-mediated repression likely involves a post-translational interaction with p28/p88 encoded by a different RNA.

### p28-GFP fusion protein forms large, mobile, intracellular inclusions that correlate with repression of TCV replication

**Fig 3:**
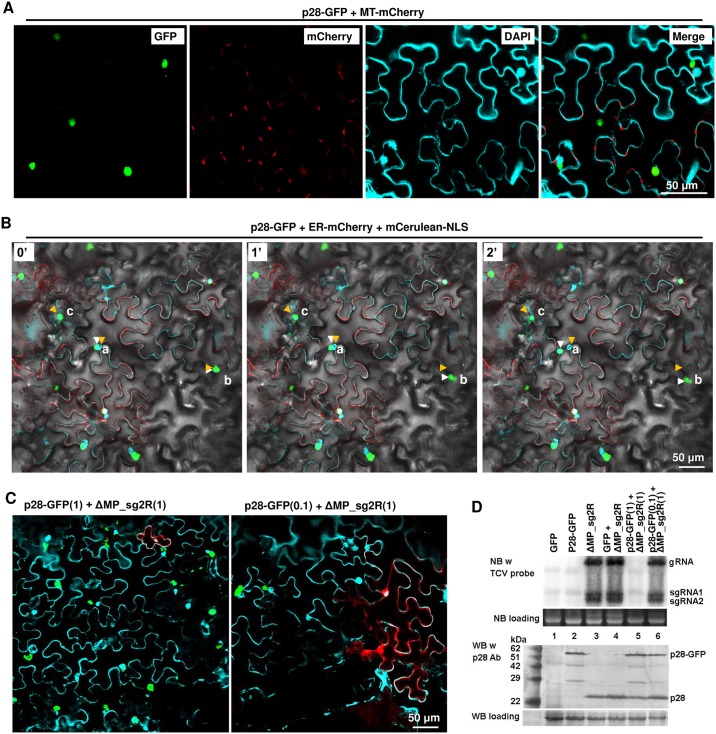
p28-GFP fusion protein form large, mobile intracellular inclusions that are not associated with cell nuclei or mitochondria, and repress the replication of a co-introduced TCV replicon. Also see S3 Fig for the spatial separation of p28-GFP foci relative from ER. A p19-expressing construct was included in all infiltrations. (A) p28-GFP forms dense foci that rival nuclei (DAPI-stained) in size, and spatially separate from mitochondria and cell nuclei. MT-mCherry: a construct expressing mCherry tagged with an N-terminal mitochondrion-localizing signal. Note that DAPI stains cell nuclei as well as cell wall. (B) Mobility of p28-GFP foci. ER-mCherry: a construct expressing mCherry tagged with an N-terminal endoplasmic reticulum-localizing signal. mCerulean-NLS: a construct expressing mCerulean tagged with a C-terminal nuclear localization signal (NLS). The images additionally contained a bright field layer to help define cell boundaries. The three panels denote the same leaf area photographed at three time points: 0, 1, and 2 minutes. Three lower case letters, a, b, and c, denote three mobile p28-GFP foci, with orange and white arrowheads marking the starting and ending points, respectively. Also see a time lapse movie (S1 Video) in Supporting Information for more details. (C) p28-GFP foci do not occur in the same cells with mCherry expressed from a co-introduced replicon (ΔMP_sg2R). The two constructs were mixed at either 1:1 (left) or 0.1:1 ratios. Only merged images were presented. (D) NB and WB verifications of results in (C). Note in lane 5 that the wild-type p28 translated from the ΔMP_sg2R replicon was readily detectable despite of its inability to replicate.

To understand how p28 represses TCV replication, we next examined its subcellular localization by expressing it as a fusion protein with GFP attached to its C-terminus (p28-GFP). As shown in S3 Fig (GFP panel), most of the transiently expressed p28-GFP within each cell coalesced into large (5–10 μm in diameter), intensely fluorescent foci. Interestingly, these foci did not co-localize with endoplasmic reticulum (ER, visualized with ER-mCherry; mCherry and Merge panels), or nuclei, or cell wall (both visualized with DAPI; DAPI and Merge panels. Note the white arrows). Since TCV replication was shown to alter the structure of mitochondria (MT) [21, 23], we further evaluated whether p28-GFP foci co-localized with MT using an MT-mCherry marker. As shown in Fig 3A, MT-mCherry appeared as tiny, elliptical dots (mCherry panel). p28-GFP foci and MT-mCherry dots were occasionally next to each other but rarely overlapped (Merge panel). Therefore, these p28-GFP foci appear to be physically distinct from sites of TCV replication.

Intriguingly, many of the p28-GFP foci underwent dynamic changes in their shape, size, and intracellular location. In Fig 3B, we highlight three of such foci, designated a, b, and c, by using orange and white arrowheads to mark their start and ending positions within a two minute timeframe. Focus a overlapped with the nucleus of the resident cell at the beginning but became clearly separated two minutes later. Similarly, focus b migrated approximately 20 μm downwards and changed shape along the way. Finally, focus c underwent similarly remarkable shape and size changes, despite of its less dramatic movement. Changes of foci a, b, and c, as well as several smaller p28-GFP foci, are further illustrated in a time lapse movie (S1 Video). Together these data clearly demonstrate that p28-GFP coalesces into highly mobile, dynamic inclusions in the cells of its expression that are likely not part of VRCs.

We next tested whether p28-GFP repressed TCV replication by co-expressing p28-GFP and ΔMP_sg2R. As shown in Fig 3C, left panel, most cells treated with both p28-GFP and ΔMP_sg2R constructs at a 1:1 ratio acquired the large irregular inclusions characteristic of p28-GFP. Only a few cells were observed to express mCherry, and these cells were always devoid of GFP, suggesting that they failed to take in the p28-GFP construct. The number of mCherry-expressing cells could be increased by reducing the p28-GFP to ΔMP_sg2R ratio to 0.1:1 (Fig 3C, right panel), and again the red fluorescent cells were free of GFP. We confirmed these findings with NB of TCV RNA, and WB of p28 protein (Fig 3D). While ΔMP_sg2R gRNA and sgRNAs were detected at similar levels in the absence or presence of unfused GFP (lanes 3 and 4), they became undetectable in the presence of p28-GFP (lane 5). Reducing p28-GFP input to one-tenth of ΔMP_sg2R largely released this repression (lane 6). Since both constructs were restricted to the cells they initially entered, these results strongly suggest that p28-GFP, similar to p28-HA, dominantly repressed ΔMP_sg2R replication in the same cells.

The 2X35S promoter-driven transcription of primary ΔMP_sg2R transcripts occurs independently of viral replication. As a result, some p28 proteins are expected to be translated from these primary transcripts even if replication does not take place. This experimental set-up provided us with the opportunity to test whether p28-GFP exerts its repressive activity by interfering with the translation of p28 from a replicon RNA. As shown in Fig 3D, when subjected to WB with a p28 antibody, leaves treated with either p28-GFP or ΔMP_sg2R alone accumulated p28-GFP and p28, respectively (ca. 55 or 28 kilo-daltons [kDa]) (lanes 2 and 3). Surprisingly however, leaves treated with both of them accumulated both proteins to easily detectable levels (lane 5). The detection of p28 in the co-inoculated leaves is particularly noteworthy because its corresponding ΔMP_sg2R transcript level was at least 10-fold lower than in cells with ΔMP_sg2R alone (compare lanes 3 and 5). Thus, p28-GFP-mediated repression of replication must have occurred at a step downstream of p28 translation. These results are consistent with those of Fig 2C implying normal translation of p88 from the repressed ΔMP_sg2G replicon. Together they indicate that p28-GFP blocked ΔMP_sg2R replication post-translationally, probably by disrupting the replication function of p28 (and p88) translated from ΔMP_sg2R RNA.

### Untagged, replication-independent p28 exhibits opposite activities depending on the co-introduced replicons

The results presented in previous sections, while consistent with a pivotal role of p28 in SIE induction, also beg the immediate question of whether p28 expressed independently of replication, without any C-terminal tags (i.e. 2XHA or GFP), still represses a TCV replicon. This question needed to be resolved because if it does, it would be puzzling that any of our replicon constructs could initiate TCV replication at all, as their primary transcripts would all be expected to translate p28 as the first protein (e.g. S1 Fig; Fig 3D). On the other hand, if it does not, then neither p28-HA nor p28-GFP would reflect an inherent function of p28, which would in turn contradict the observation that the p28-only defective replicon (Δp88ΔMP_sg2R; Fig 2C) could repress a different replicon (ΔMP_sg2G; Fig 2C).

**Fig 4:**
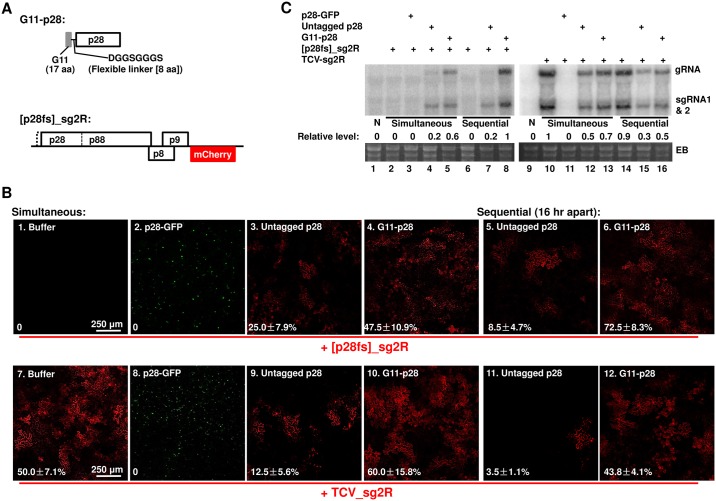
Compared to the N-terminally tagged G11-p28, untagged p28 is less competent at complementing the replication of a p28-defective replicon, but more potent at repressing the replication of another replicon encoding wt p28. (A) Diagrams of the N-terminally tagged G11-p28 construct, and the p28-defective [p28fs]_sg2R replicon. (B) Complementation of [p28fs]_sg2R (top row), and repression of TCV_sg2R, by various p28 derivatives (p28-GFP, untagged p28, and G11-p28). As in earlier experiments, all treatments included a p19-expressing construct. Within each row, the left four panels represent leaf patches infiltrated with transiently expressed p28 derivatives and replicon constructs simultaneously. By contrast, the right two panels represent leaf patches that were first infiltrated with p28 derivatives, and then with replicons, with a 16 hour delay. The numbers on the panels represent the averaged percentages of cells with mCherry fluorescence indicating active replication, plus SDs.(C) NB confirmation of results in (B). The relative accumulation levels of replicon genomic RNA were estimated with ImageJ. Note that the readings of lane 8 and 10 were set at 1 for the left and right blots, respectively.

**Fig 5:**
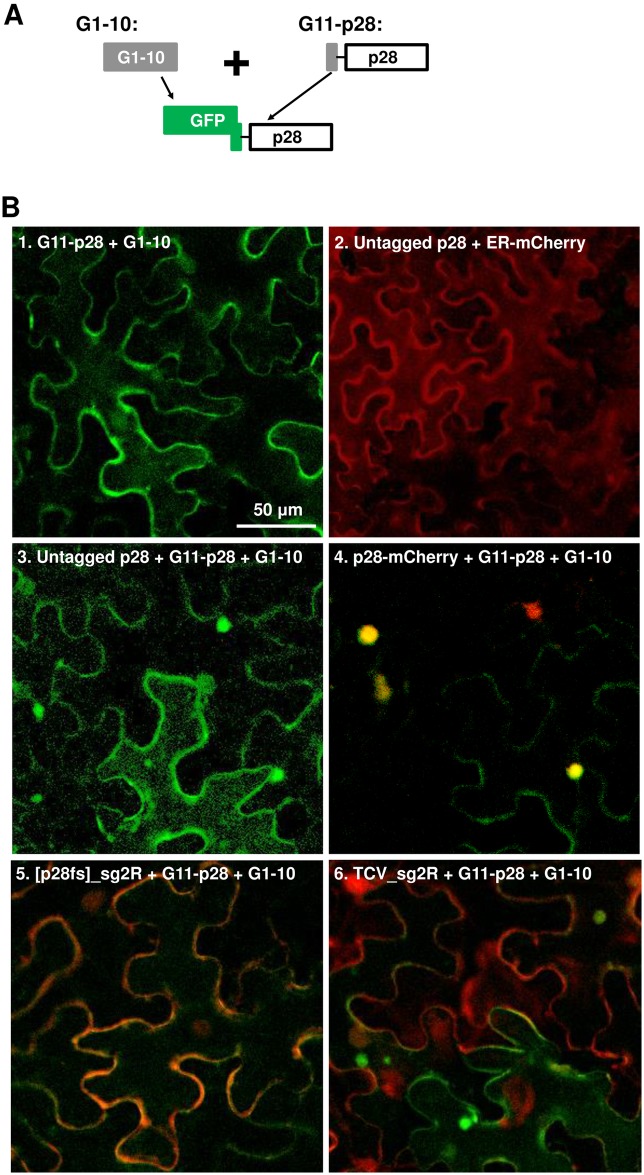
The G11-p28 derivative of p28 does not form intracellular inclusions, but is *trans*-coalesced by untagged p28 and p28-mCherry. As in previous experiments, a p19-expressing construct was present in all infiltrations. (A) Diagram of the constructs used. Note that the N-terminal G11 tag of G11-p28 does not fluoresce by itself, but becomes fluorescent in the presence of G1-10 expressed from a separate construct. (B) Confocal microscopy of various constructs expressed in *N*. *benthamiana* cells. Only merged images were presented. Bar = 50 μm. See time lapse movies in S2 and S3 Videos for the intracellular migration of the p28 inclusions induced by replicon-borne p28.

To resolve this puzzle, we produced three new constructs: the first would express an untagged p28 in agro-infiltrated cells. The second, G11-p28, should express a p28 variant tagged at the N-terminus with a 25-aa “G11” tag derived from the 11^th^ β-strand of GFP (Fig 4A). The value of G11 tag will become apparent later (Fig 5). Note that these two constructs express the untagged p28 and G11-p28 proteins independent of TCV replication. Finally, the third construct, [p28fs]_sg2R, harbors a non-replicating TCV mutant that contains a one-nt deletion at position 106, causing p28 (and p88) translation to stop after 14 aa. However, the p88 function of [p28fs]_sg2R was little affected, as the replication of this mutant was restored by providing only the p28 (Fig 4A and 4B). An N-terminally truncated p88 could be produced from [p28fs]_sg2R through translational re-initiation at another AUG codon 36 aa downstream.

Contrary to the prevailing assumption that replication function of p28 is *cis*-acting [22], the transiently expressed, untagged p28 was able to partially complement the replication of the p28-defective [p28fs]_sg2R. As shown in Fig 4B, [p28fs]_sg2R did not replicate by itself (panel 1; also Fig 4C, lane 2), but replicated in an average of 25% cells in the presence of untagged p28 (panel 3; Fig 4C, lane 4). Notably, no complementation was observed with p28-GFP (Fig 4B, panel 2), suggesting that the C-terminal GFP tag abolished the replication function of p28. Unexpectedly, the N-terminally tagged G11-p28 complemented the p28-defective replicon twice as efficiently as p28 (compare panels 3 and 4 of Fig 4B, and lanes 4 and 5 of Fig 4C), suggesting that the N-terminal G11 tag rendered p28 more replication-active, possibly by overcoming certain repressive state of p28 (see later). Importantly, complementation by untagged p28 weakened only modestly when [p28fs]_sg2R was introduced with a16-hour delay (Fig 4B, panel 5; and Fig 4C, lane 7), suggesting that the replication state of p28, once established, was minimally affected by extended pre-accumulation. By contrast, complementation by G11-p28 actually strengthened when the defective replicon was delayed, further confirming G11-p28 as more replication-active than untagged p28 (compare Fig 4B, panels 4 and 6; Fig 4C, lanes 5 and 8).

However, when co-introduced with the wild-type (wt) p28-encoding TCV_sg2R replicon, transiently expressed p28 turned repressive. As shown in Fig 4B, untagged p28 caused the number of red fluorescent cells to decrease to about 1/4 of the TCV_sg2R only control (compare panels 7 and 9), indicating a partial repression of the replicon by untagged p28. Furthermore, this repression by p28 became more potent when the TCV_sg2R replicon was introduced with a 16-hour delay, reducing the number of cells with replication-dependent mCherry fluorescence to 1/15 of the control (panel 11). Notably, the N-terminally tagged G11-p28 was substantially less repressive than untagged p28 (Fig 4B, panels 10 and 12). This contrasts with the C-terminally tagged p28-GFP that caused a complete loss of TCV_sg2R replication (Fig 4B, panel 8; also Figs 2 and 3), suggesting that these tagged forms of p28 represent two extremes of the repressive activity of wt p28.

These data were further corroborated with NB (Fig 4C). The reduction in TCV_sg2R RNA levels caused by untagged p28 was less dramatic than the numbers of red fluorescent cells, possibly reflecting the limitation of confocal microscopy in detecting cells with very low mCherry expression (Fig 4B and 4C). Together these results strongly suggest that transiently expressed, untagged p28 exists in two different states—one replication-active, the other repressive. Importantly, the replication-active state, once established early on, did not readily transit to the repressive state, as evidenced by the sustained ability of p28 (and G11-p28) to complement sequentially delivered [p28fs]_sg2R (16 hour delay; Fig 4B, panels 3 and 5). Intriguingly, the repressive state of p28 that repressed the co-introduced TCV_sg2R must have established itself fairly early as well. The co-existence of two p28 states with opposite functions in turn suggests that their corresponding protein fractions probably exist in separate cellular compartments.

### Both transiently expressed and replicon-borne p28 converts the diffuse G11-p28 to punctate inclusions

Results presented above strongly suggest that untagged p28 exists in both replication-active and –repressive states. Since the replication-repressing p28-GFP formed large, dynamic, and mobile intracellular inclusions, we next wondered if the repressive state of untagged p28 also existed in similar inclusions. Because untagged p28 does not fluoresce by itself, we first determined whether G11-p28, being replication-competent but repression-deficient, were capable of forming punctate inclusions. Detection of G11-p28 was in turn facilitated by co-expression of G1-10, the first 10 β-strands of GFP, which interacts with the G11 tag to generate green fluorescence (Fig 5A) [29, 30]. As shown in Fig 5B, panel 1, GFP fluorescence reconstituted by G11-p28 and G1-10 co-expression showed a clearly diffuse distribution without discrete foci. Since G11-p28 efficiently complemented the replication of a p28-defective mutant (Fig 4), this result suggests that the replication function of p28 does not require the formation of punctate foci.

In contrast, while untagged p28 alone did not cause detectable changes in cell morphology (panel 2, cell boundaries visualized with ER-mCherry), its co-expression with (G11-p28 + G1-10) led to the coalescence of green foci in approximately 70% of fluorescent cells, and a reduction of diffuse GFP in the same cells (Fig 5B, panel 3; note the top, left, and right cells contained green foci but little diffuse green fluorescence, whereas the bottom middle cell contained mostly diffuse green fluorescence). We infer that these intensely fluorescent foci must have arisen from the coalescence of the otherwise diffuse G11-p28, induced by the co-expression of untagged p28. This in turn suggests that untagged p28 could self-associate into punctate inclusions capable of coalescing G11-p28. Consistent with this inference, p28-mCherry, which like p28-GFP formed visible inclusions, coalesced G11-p28 much more efficiently than untagged p28, leading to dominance of mostly yellow foci in co-infiltrated cells, and concomitantly near complete loss of diffuse G11-p28 (Fig 5B, panel 4; note yellow foci). Together these results reveal two important insights: (i) transiently expressed, untagged p28 induced the formation of punctate inclusions in a substantial fraction of cells; and (ii) the p28 (and p28-mCherry) inclusions, once formed, are capable of seeding the coalescence of a non-coalescing p28 mutant (G11-p28).

Notably, complementation of the p28-defective [p28fs]_sg2R by G11-p28 (plus G1-10) was accompanied by diffuse distribution of both GFP and mCherry, resulting in cells that emit diffuse, brown fluorescence (Fig 5B, panel 5). By contrast, co-expression of G11-p28 (plus G1-10) with TCV_sg2R encoding wt p28 led to the appearance of green foci in approximately 50% of cells, indicating that wt p28 expressed from the replicon RNA likewise formed punctate inclusions that seeded G11-p28 coalescence (Fig 5B, panel 6). Strikingly, some of the focus-harboring cells contained little or no red fluorescence, suggesting that in these cells the replication-repressive state of p28 was established very early, thus preventing the initiation of replication by TCV_sg2R. This outcome mirrors the inefficient replication initiation by TCV replicons documented in S1B Fig, suggesting that at least in some cells, p28 translated from replicon transcripts turned repressive before the transcripts had chance to commence replication. Most importantly, similar to p28-GFP inclusions, these replicon-induced inclusions were highly mobile, often racing across the entire cell in less than ten minutes (time lapse S2 and S3 Videos in Supporting Information).

Here we summarize several key observations concerning the role of p28-induced punctate inclusions in SIE. (i) The inclusions are not required for replication function of p28. (ii) Untagged p28, expressed either transiently or replication-dependently, is capable of forming inclusions, though less efficiently than C-terminally tagged p28 derivatives. (iii) Once formed, these inclusions nucleate the otherwise non-aggregating p28 derivatives. (iv) The inclusions are highly mobile, likely enabling them to capture monomeric p28, sequestering them from the alternative functionality of p28 (replication). Together they support the concept that these inclusions correspond to a repressive state of p28, and they exert the repressive function by trapping freshly translated p28 molecules.

### Replicon-expressed G11-p28 supports replication, but not SIE

**Fig 6:**
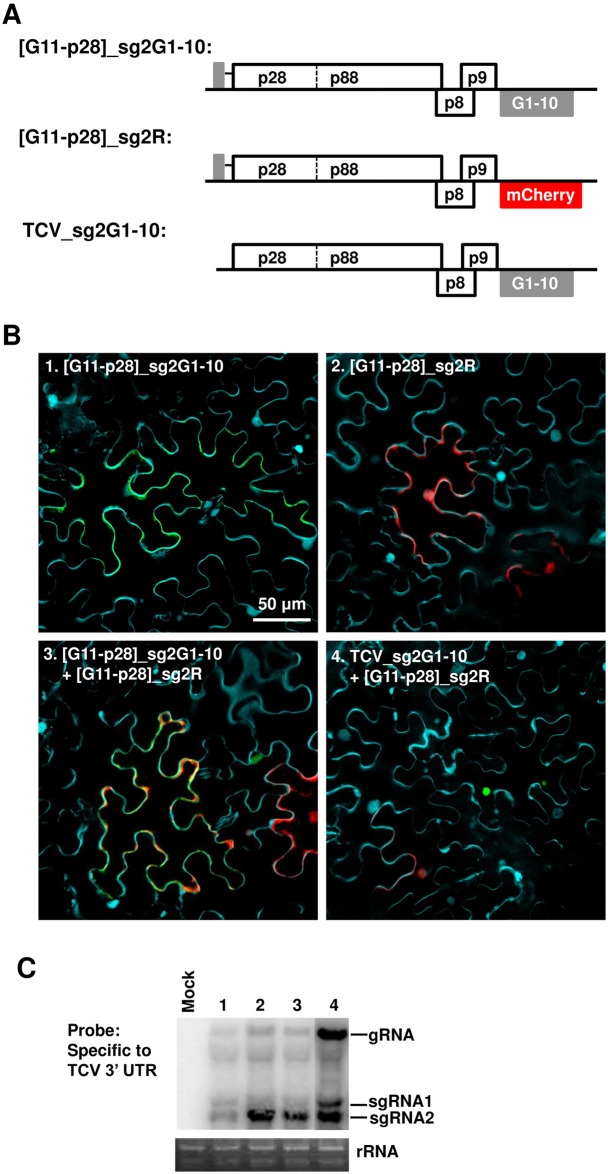
TCV replicons encoding G11-p28 abolish SIE among themselves, but are dominantly repressed by those expressing wt p28. (A) Diagrams of three replicon constructs used in the current set of experiments. Note that GFP fluorescence reconstitution by the top construct is dependent on its successful replication, and fluorescence signals co-localize with G11-p28. By contrast, mCherry expressed from the middle construct, while dependent on replication as well, should not associate with G11-p28. (B) Confocal microscopy of the replicon constructs expressed either separately or in combination in *N*. *benthamiana* cells. Only merged images were presented. (C) Verification of replication of various TCV replicons with NB.

Having shown that the replication-competent, yet repression-deficient G11-p28 by itself maintained a diffuse distribution, but was converted to punctate inclusions by p28, we next asked whether G11-p28 expressed from a replicon could support TCV replication and SIE. To address this question, we generated two new replicon constructs (Fig 6A). The first, [G11-p28]_sg2G1-10, fused G11 to the N-terminus of p28, and incorporated G1-10 in the sgRNA2 coding region. Since sgRNA2 can only be synthesized following successful replication, the expression of G1-10 from this replicon, and resulting green fluorescence, will only occur if G11-p28 can function in replication. The second construct, [G11-p28]_sg2R, was identical to the first except that G1-10 was replaced by mCherry (Fig 6A).

When introduced individually into cells, the [G11-p28]_sg2G1-10 and [G11-p28]_sg2R replicons each produced fluorescence in approximately 10% of cells (Fig 6B, panel 1 and 2), suggesting that the G11 insertion compromised, but did not abolish, the replicability of the replicons. This was also confirmed with NB (Fig 6C, lanes 1 and 2). As expected, the green fluorescence reconstituted by G11-p28 and G1-10 co-expression was diffusely distributed (Fig 6B, panel 1). To our surprise, co-introducing these two constructs into the same cells led to approximately 50% of fluorescent cells to express both GFP and mCherry, indicating a loss of SIE that allowed the simultaneous replication of both the [G11-p28]_sg2G1-10 and [G11-p28]_sg2R replicons. We conclude that the replicon-borne G11-p28 was incapable of exerting SIE, despite of retaining p28’s activity in replication. Hence, the SIE function of p28 in the replicon background was likewise successfully decoupled from its replication function by the N-terminal G11 tag.

### Replicon-expressed wt p28 exerts SIE to a G11-p28-expressing replicon, and seeds G11-p28 coalescence

Our data thus far revealed that G11-p28 simultaneously weakens p28’s propensity to form inclusions and compromise its ability to exert SIE, leading us to hypothesize that the p28 inclusions are directly responsible for SIE. To further test this hypothesis, we asked if untagged, wt p28 expressed from a TCV replicon could exert dominant SIE to another replicon encoding G11-p28. To do so, we further generated the TCV_sg2G1-10 replicon that encoded wt p28 but expressed G1-10 from sgRNA2, which could not fluoresce unless G11-p28 translated from a different construct is present in the same cell (Fig 6A). *N*. *benthamiana* leaf cells were then treated with a mixture of TCV_sg2G1-10 and [G11-p28]_sg2R constructs. As expected, most of the fluorescent cells contained green but not red fluorescence (Fig 6B, panel 4), demonstrating preferential replication of TCV_sg2G1-10 encoding wt p28, and simultaneous repression of [G11-p28]_sg2R encoding G11-p28 (which nevertheless could translate G11-p28 independent of replication). Importantly, the green fluorescence existed as dense foci in these cells, confirming the coalescence of G11-p28 through an interaction with wt p28 that exerted SIE to [G11-p28]_sg2R. Although a few isolated cells showed red fluorescence indicative of [G11-p28]_sg2R replication (Fig 6B, panel 4, lower left), these cells never contained any GFP fluorescence, suggesting that they took in only the [G11-p28]_sg2R construct. Together these data further support the idea that a repressive state of p28 exerts SIE by *trans*-aggregating fresh, diffuse form of p28 translated from superinfecting TCV genomes.

## Discussion

SIE acts in the cells already occupied by a virus to deny the chance of secondary infections by the same or closely related viruses, but has no effect on more distantly related viruses. While SIE has been observed in infections of a wide range of viruses with diverse genome structures and host tropisms, its molecular mechanism remain poorly understood. Studies using CTV (different from TCV!) found that SIE can be abolished by replacing three virus-encoded proteins, namely the replication-related L1 and L2 proteases, as well as movement-related p33, with their counterparts from a distantly related CTV strain [8, 31]. Similarly, SIE of WNV and HCV was found to be reversed by mutations in a few viral proteins, among them WNV-encoded 2K and NS4A, and HCV-encoded E1, p7, and NS5A [4, 19]. More recently, Tatineni and French [20] revealed that proteases and capsid proteins encoded by two plant-infecting potyviruses were responsible for SIE of cognate viruses. These studies suggest that SIE is exerted by a relatively few proteins encoded by the primary virus. Mechanistic dissections using several (+) ssRNA animal viruses (WNV, HCV, and BVDV) further suggest that SIE functions primarily by blocking the replication of the superinfector [2–4, 19]. However, exactly how this blockage is achieved remains a mystery.

### Mechanism of SIE in TCV infections

**Fig 7:**
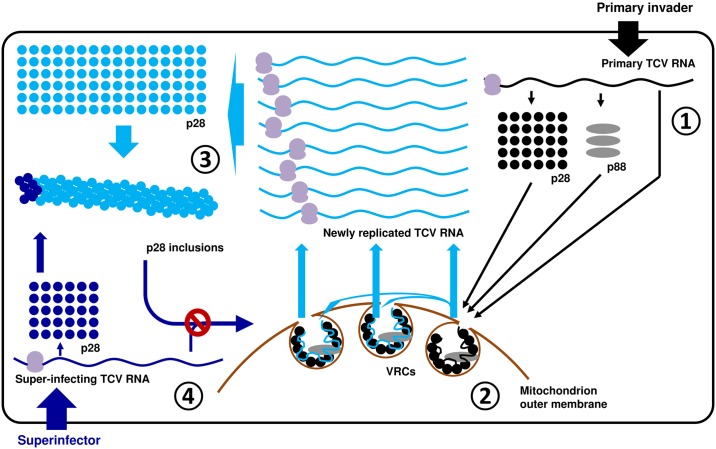
A model of TCV p28-mediated SIE. Step 1: the concentration of p28 translated from the genome of primary invader (top right, black dots) is relatively low. They interact with each other relatively slowly, allowing the incorporation of p88, and enclosure of the complex by mitochondrion outer membrane to form VRCs. Step 2: genomic RNA of the primary invader enters VRCs to initiate replication. We hypothesize that some VRCs stay vacant and await the entry of newly synthesized viral RNA. However, the majority of progeny RNA will probably not have the chance to repeat the replication cycle in the same cell. Also beginning with this step, large amounts of progeny RNA become template for p28 translation, drastically increasing the intracellular concentration of p28. Step 3: higher p28 concentration propels faster p28 polymerization, leading to quick formation of oversized p28 polymers that escape the membrane enclosure. Note that the forms of polymeric p28 complexes are hypothetical as we do not yet know the specific mode of p28-p28 interactions. Step 4: since the p28 polymers are by definition capable of nucleating homologous monomers, they are expected to capture the p28 monomers (blue dots) translated from superinfector genomes, preventing them from forming new VRCs.

In the current study, we demonstrate that SIE among TCV variants is adequately explained by the action of p28, one of the TCV-encoded replication proteins, that exists in two distinct functional and structural states in infected cells. Collectively our data support a model through which p28 acts both as a replication facilitator and a repressor, depending on its cellular concentration (Fig 7). First and foremost, we hypothesize that both the replication and SIE functions of p28 derive from its inherent ability to self-interact to form oligomers. These oligomers become seeds onto which additional p28 molecules are coalesced, ultimately leading to structures responsible for replication or SIE. At the very early stage of a TCV invasion when p28 concentration is relatively low, the rate of p28 polymerization is slow, as the polymerization itself necessarily lowers p28 concentration in the cytoplasm. This rate is probably further constrained by various steps of virus replication complex (VRC) formation, including incorporation of p88 and/or viral RNA into p28 polymers, and enclosure of the complex by mitochondrion outer membrane [21, 23] (Fig 7, steps 1 and 2). On the other hand, in cells where a resident TCV is undergoing active replication, the large amounts of progeny TCV genomes template p28 translation to very high levels. This could in turn accelerate the polymerization of p28, leading to the formation of oversized p28 complexes that escape membrane enclosure, permitting easy trapping of freshly translated p28 monomers, including those translated from superinfecting TCV genomes, thus preventing the formation of new VRCs, leading to SIE (Fig 7, steps 3 and 4). Note that the structures of p28 complexes depicted in Fig 7 are hypothetical as we currently do not know their structural details. It is further possible that high levels of p28 might also dilute out the host proteins needed for VRC assembly, thus favoring the coalescence of p28 inclusion bodies.

Importantly, the formation of large cytoplasmic p28 complexes does not prevent the p28 molecules already sequestered inside VRCs from functioning in viral replication. This arrangement ensures that the primary invader continues to replicate inside VRCs, while superinfectors are subdued by the cytoplasmic p28 complexes. That p28 can exist both in membrane-associated VRCs and repressive cytoplasmic complexes is not unprecedented, as similar partitioning was reported for the tomato mosaic virus (ToMV)-encoded 130K replication protein, which exists in a membrane-associated, replication-competent form, as well as a cytoplasmic form capable of suppressing RNA silencing [32–34].

Our model is highly consistent with the observation that the strongly SIE-inducing p28 variants, including p28-GFP/mCherry/HA, form large, dynamic, and mobile cytoplasmic inclusions that trans-aggregate the otherwise diffusely distributed, yet replication-active G11-p28. Furthermore, our experiments indirectly implicated untagged p28, expressed either transiently or during replication, in similar cytoplasmic inclusions. It can be envisioned that the C-terminal tags could enhance the stability of p28 oligomers, thus favoring the formation of oversized polymers, whereas the N-terminal G11 tag could destabilize p28 oligomers, thus slowing down the polymerization.

Our model provides an immediate explanation for the inefficient initiation of replication by agro-delivered replicon constructs (S1B Fig). The strong 2X35S promoter in these constructs drives the transcription of primary TCV RNA to levels that are higher than natural viral invasions (typically a few viral genomes), but lower than progeny genomes produced by active replication. As a result, p28 oligomerization likely takes place at an intermediary rate that blocks most primary TCV transcripts from replication, yet allows occasional escape from this blockage in a stochastic manner (S1B Fig). Our model further asserts that the ones that do evade this blockage would in turn produce vastly more abundant p28, thus solidifying the repression against other co-introduced replicons.

### Are p28-containing cytoplasmic inclusions VRCs?

Previous studies of other virus models appear to link large cytoplasmic inclusions formed by viral replication proteins with active replication [35, 36]. Based on our results, this view might warrant a second look. Specifically, we show that the N-terminally tagged G11-p28 robustly complemented the replication of a p28-defective replicon, yet did not form large cytoplasmic inclusions, and was incapable of SIE. By contrast, the C-terminally tagged p28-GFP/mCherry and p28-HA were completely inactive at replication, but formed large inclusions and exerted powerful SIE. These results suggest that the cytoplasmic inclusions represent the repressive form of p28, and that replication-active VRCs might be rather orderly structures discernable only with electron microscopy [37]. Presence of viral RNA in the cytoplasmic foci does not necessarily signal active replication, as viral RNA could become trapped in the foci through interactions with p28 and its analogs. Finally, our results also call for caution when interpreting experiments using tagged proteins, as these tags could alter the balance among various functional state(s) of original proteins.

### SIE as the underlying mechanism for *cis*-preference of p28

Many virus-encoded replication proteins are *cis*-acting or at least *cis*-preferential, meaning they exclusively or preferentially benefit the very viral RNA molecule from which they are translated [22, 38–41]. In most (+) ssRNA viruses, the *cis*-acting protein typically corresponds to the replication protein that is more highly expressed, yet does not possess RNA-dependent RNA polymerase (RdRP) signature, thus analogous to p28 of TCV. Kawamura-Nagaya and colleagues [40] found that the replication-competent form of TMV p126 bound to TMV genomic RNA co-translationally, thus proposing this p126-RNA binding as the underlying mechanism for p126 *cis*-preference. However, it is uncertain as to how widely applicable this mechanism is, as it does not explain how the 1a protein encoded by the tripartite brome mosaic virus (BMV), being *cis*-preferential relative to the 1a-coding RNA1, is nevertheless able to function in *trans* to facilitate the replication of the other two BMV genome segments (RNA2 and RNA3) [39].

Similar to TMV p126 and BMV 1a, TCV p28 is also known to be *cis*-preferential [22] (also see Fig 2C). However, our new findings suggest that *cis*-preference of p28 likely reflects a strong *trans*-repression rather than *cis*-action. We show that transiently expressed p28 complemented a mutant replicon incapable of producing its own wt p28. Therefore, p28 *cis*-preference is readily overcome once the following two conditions are met: (i) p28 is expressed from a non-replicating mRNA; and (ii) the defective replicon does not encode a functional p28. It should be noted that this experimental system differs from that of White and colleagues [22], where the two mutant replicons used, ΔAPA and RT, produced either p28 (ΔAPA) or p88 (RT), but not both. As a result, the preferential amplification of the p28-encoding ΔAPA replicon could be explained by the fact that the p28 produced by a replicating ΔAPA could strongly repress the p88-encoding RT, whereas p88 produced by the RT mutant would be only weakly repressive (e.g. Fig 2A). This difference in repressive activity between p28 and p88 could cause p28 to appear to be *cis*-preferential. While it remains to be determined whether this mechanism applies to *cis*-acting proteins of other viruses, we note that it allows the replication-active form of p28 (and its analogs in other viruses) to be accessed by non-p28-coding RNAs like satellite RNAs, defective interfering RNAs and, in viruses like BMV, other genome segments. Of course, this model does not rule out the possibility that certain *cis*-acting RNA elements could function to de-aggregate p28 complexes, thus permitting their re-entry into the replication-competent pool.

### SIE as the underlying mechanism for viral population bottlenecking at the cellular levels

Multi-variant populations of many plant viruses are known to undergo dramatic bottlenecking during systemic infections [28, 41–44]. Recent studies strongly suggest that this population bottlenecking is likely caused by cellular level SIE exerted by viral variants that arrive at certain parts of the plants (e.g. vascular bundles and systemic leaves) ahead of other variants [17]. The SIE mechanism proposed in this study additionally provides an alternative explanation for the intracellular population bottlenecking observed by Miyashita and colleagues [45]. These authors found that individual tobacco cells receiving a viral population comprising thousands of ToMV variants allowed a tiny fraction of the incoming variants (fewer than 10) to replicate. The authors invoked active but stochastic degradation by host cell nucleases as a possible reason of observed population bottlenecking [45]. Based on the SIE mechanism proposed here, we think that it is likely that, as soon as a few variants start to replicate, they would swiftly produce large amounts of replication-repressing viral proteins (or proteins like p28 that exert both replication and repression functions) that actively block the replication of other variants. This model is further consistent with the authors’ observation that the handful surviving variants in each cell accumulated varying numbers of progenies [45]–these variants could have initiated replication at different post-entry time points prior to the full establishment of the repressive state.

### Adaptive rationale of the proposed SIE mechanism

We noted earlier that SIE was observed in diverse viruses with distinct genome structures and host tropisms, suggesting it as an evolutionarily advantageous trait for viruses. With our current understanding of the TCV system as the basis, we next consider the underlying rationale for the preservation of SIE by viruses. In our model, we hypothesize that p28 translated from the newly replicated TCV genomes mostly serves to reinforce the repressive state of p28. The logical extrapolation of this idea is that these newly replicated TCV RNAs are also targeted by the repressive state of p28. Indeed, we speculate that they are the primary or “intended” target—the superinfector genome becomes targeted because it is “mistaken” as one of the newly synthesized viral RNAs. Why is then a trait that represses the replication of progeny genomes selected for? We reason that such a trait would ensure random mutations accidentally incurred during the viral replication process are isolated in a minimal number of progeny genomes. This is particularly important for RNA viruses as virus-encoded RdRPs are known to be error-prone, estimated to introduce close to one error per genome per replication cycle for TCV-sized genomes [46]. Consequently, allowing progeny genomes to repeat the replication cycle inside the cells of their origin would not only cause the proliferation of these errors, but also exacerbate their potential damages by compounding them with additional errors. By denying the progeny genomes the chance to re-replicate, SIE constitutes a powerful adaptive constraint that maximizes the genome stability of RNA viruses, and possibly also viruses with DNA genomes.

In summary, the current study reveals a novel mechanism of SIE that engages a repressive state of a replication protein to capture its counterparts translated from superinfected (and newly synthesized) viral genomes, thereby preventing the latter from entering replication cycles. While substantial additional research is needed to resolve the detailed structure and functional mode of the repressive form of the protein involved, we are confident that this general mechanistic framework will prove to be applicable to other viruses, possibly leading to the development of novel antiviral strategies that target this mechanism.

## Materials and methods

### Constructs

The TCV_sg2G construct was from a previous study (formerly named TCV-GFP) [47, 48]. Briefly, this construct is based on a binary plasmid (pPZP212) that replicates in both *E*. *coli* and *Agrobacterium tumefaciens* (strain C58C1). The TCV cDNA, in which the 5’ 2/3 of the CP coding sequence was replaced by that of GFP, was placed under control of 2X35S to permit the transcription of primary TCV RNA by RNA polymerase II of plant cells. Both TCV_sg2R and CarMV_sg2R were produced in a similar manner. Note that the mCherry coding sequence was modified to eliminate sites of common restriction enzymes while maintaining the amino acid sequence (the modified mCherry sequence, designated mCherry2, is available upon request). ΔMP_sg2G and ΔMP_sg2R were derivatives of TCV_sg2G and TCV_sg2R, respectively, that incorporated a 92-nt deletion within the MP coding region [26, 49]. Δp88Δ92_sg2R was in turn a derivative of ΔMP_sg2R that contained a 4-nt deletion within the p88 coding region, as a result of digestion with ApaI, followed by blunt-ending and relegation [22].

Replicon constructs containing G11-tagged p28 were generated through a three-step manipulation. First, in order to maximize the number of unique restriction enzyme sites, the 2X35S-TCV_sg2R-T35S cassette was switched to pAI101, a pCambia1300 derivative with a modified multiple cloning site, to create pAI-TCV_sg2R. Next, to produce [G11-p28]_sg2R, a gBlock fragment (IDT, Coralville, IA) consisting of TCV 5’ UTR plus the p28 start codon (66 nt), the coding sequence of the 11^th^ β-strand of GFP connected to an 8-aa flexible linker (GRDHMVLHEYVNAAGITDGGSGGGS), and the first 63-nt of p28 coding sequence was synthesized, and used to replace the first 129 nt of TCV cDNA (released by XhoI and XmaJI sites) in pAI-TCV_sg2R using Gibson Assembly cloning (NEB, Ipswich, MA). Finally, the [G11-p28]_sg2G1-10 construct was created by replacing the mCherry coding sequence with that of the first 10 GFP β-strands (G1-10) [31].

Constructs 2X35S-GFP and 2X35S-p19 were described in an earlier study [27]. Other transient expressing constructs were produced similarly. The ER-targeting signal was derived from mGFP5 [50]. The mitochondrion-targeting signal was derived from the yeast cytochrome *c* oxidase IV [51].

### Agro-infiltration

Agro-infiltration experiments were conducted as described in ref [27].

### Microscopy

Confocal microscopic observations were carried out using a Leica Confocal microscope (TCS SP5) available through Molecular and Cellular Imaging Center at the Ohio Agricultural Research and Development Center, The Ohio State University.

### Quantification of fluorescent cells

The quantitative data in S1B Fig were obtained by counting fluorescent cells in viewing fields containing approximately 50 cells. For each treatment, five such fields were counted and the percentages of cells with GFP or mCherry fluorescence were calculated. The numbers presented were midpoint values with range of variations. For all other figures, the fluorescent cells were quantified as a percentage of the total number of cells in a given viewing field by dividing a minimally magnified (10X) image of the viewing field to 100 (10 X 10) equal-sized mini-squares using the View/Show/Grid option of Photoshop, and counting the number of mini-squares within each image that are at least 50% fluorescent. For each treatment, at least four images were quantified in this manner and the range of variations was provided in the relevant figures.

### Northern and Western Blotting (NB and WB)

NB and WB were carried out as described [27, 47]. A ^32^P-end labelled oligo complementary to the 3’ UTR of TCV was used to detect TCV genomic as well as subgenomic RNAs. A p28 antiserum was used to detect p28 protein in Western blots. A GFP antibody (Life Tech, Carlsbad, CA) was used to detect both GFP and mCherry.

## Supporting information

S1 Fig Delayed initiation of replication of a TCV replicon introduced to *N*. *benthamiana* cells using agro-infiltration. (A) Diagrams of constructs used in this set of experiments. (B) Comparison of the expression kinetics of transiently expressed GFP (2X35S-GFP) and replication-dependent mCherry (ΔMP_sg2R) in cells co-resided by both constructs. Note that a p19-expressing construct was included in this and subsequent experiments to protect the transcribed RNAs from RNA silencing-mediated degradation. Numbers below each panel are the averaged percentages of cells showing GFP (G) or mCherry (R) fluorescence at the respective times points. See Materials and methods for details of quantification. (C) Western blot detection of GFP and mCherry in tissues expressing the constructs indicated on the left. (D) Delayed delivery does not compromise replicon replication or SIE. *Agrobacterium* suspensions containing TCV_sg2G and TCV_sg2R were mixed and delivered into *N*. *benthamiana* cells 16 hours after an initial agro-infiltration with a p19-expressing construct.
(PPTX)

S2 Fig (A) WB confirmation of expression of HA-tagged TCV proteins. (B) Separate channel images of *N*. *benthamiana* leaf cells agro-infiltrated with a mixture of three constructs: Δp88ΔMP_sg2R + ΔMP_sg2G + p19.
(PPTX)

S3 Fig p28-GFP foci do not co-localize with ER or cell nuclei. ER network was labelled with an ER-mCherry construct, and the cell nuclei (and cell wall) were stained with DAPI. Note the arrows in the Merge panel highlight the separation of p28-GFP foci from nuclei in two different cells.
(PPTX)

S1 Video A time lapse video recording the movement of p28-GFP foci in Fig 3B. (MP4)

S2 Video A time lapse video showing the movement of p28-induced foci in replicon-infected cells (related to Fig 5B, panel 6). (MP4)

S3 Video Another time lapse video showing the movement of p28-induced foci in replicon-infected cells (related to Fig 5B, panel 6). (MP4)
