# Supplementary material for: A self-perpetuating repressive state of a viral replication protein blocks superinfection by the same virus
Source: PLoS Pathog. 2017 Mar 7;13(3):e1006253. doi: 10.1371/journal.ppat.1006253 (PMC5357057; doi:10.1371/journal.ppat.1006253)
Supplement: S1 Fig — (A) Diagrams of constructs used in this set of experiments. (B) Comparison of the expression kinetics of transiently expressed GFP (2X35S-GFP) and replication-dependent mCherry (ΔMP_sg2R) in cells co-resided by both constructs. Note that a p19-expressing construct was included in this and subsequent experiments to protect the transcribed RNAs from RNA silencing-mediated degradation. Numbers below each panel are the averaged percentages of cells showing GFP (G) or mCherry (R) fluorescence at the respective times points. See Materials and methods for details of quantification. (C) Western blot detection of GFP and mCherry in tissues expressing the constructs indicated on the left. (D) Delayed delivery does not compromise replicon replication or SIE. Agrobacterium suspensions containing TCV_sg2G and TCV_sg2R were mixed and delivered into N. benthamiana cells 16 hours after an initial agro-infiltration with a p19-expressing construct. (PPTX) [file ppat.1006253.s001.pptx]

## Slide 1
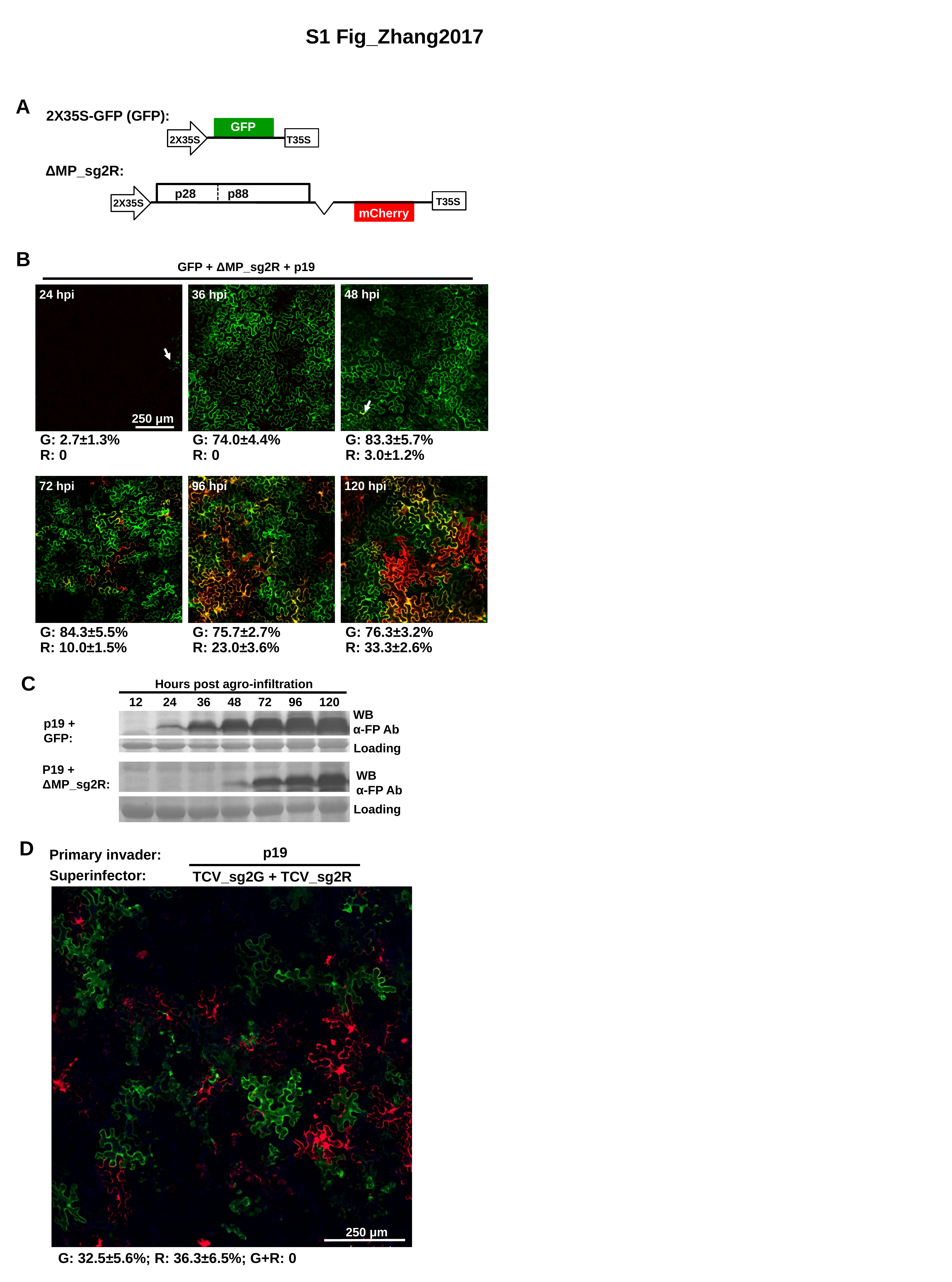

S1 Fig_Zhang2017
A
2X35S-GFP (GFP):
GFP
2X35S
T35S
ΔMP_sg2R:
p28
p88
2X35S
T35S
mCherry
B
GFP + ΔMP_sg2R + p19
48 hpi
24 hpi
36 hpi
250 μm
G: 2.7±1.3%
R: 0
G: 74.0±4.4%
R: 0
G: 83.3±5.7%
R: 3.0±1.2%
72 hpi
96 hpi
120 hpi
G: 84.3±5.5%
R: 10.0±1.5%
G: 75.7±2.7%
R: 23.0±3.6%
G: 76.3±3.2%
R: 33.3±2.6%
C
Hours post agro-infiltration
12 24 36 48 72 96 120
WB
α-FP Ab
p19 + GFP:
Loading
P19 + ΔMP_sg2R:
WB
α-FP Ab
Loading
D
p19
Primary invader:
Superinfector:
TCV_sg2G + TCV_sg2R
250 μm
G: 32.5±5.6%; R: 36.3±6.5%; G+R: 0
