# Supplementary figures and images for: A self-perpetuating repressive state of a viral replication protein blocks superinfection by the same virus
Source: PLoS Pathog. 2017 Mar 7;13(3):e1006253. doi: 10.1371/journal.ppat.1006253 (PMC5357057; doi:10.1371/journal.ppat.1006253)

## Slide 1
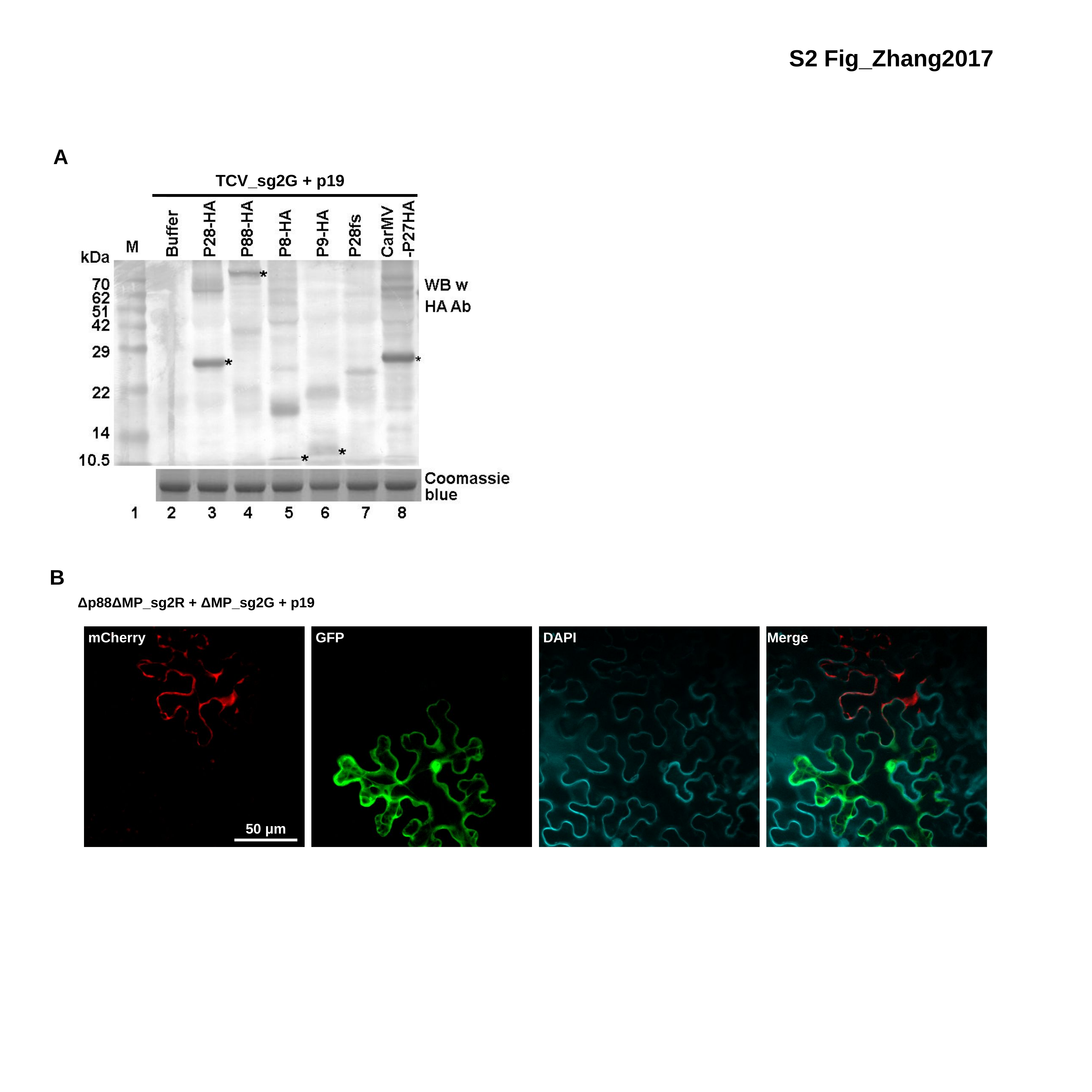

S2 Fig_Zhang2017
A
TCV_sg2G + p19
B
Δp88ΔMP_sg2R + ΔMP_sg2G + p19
mCherry
GFP
DAPI
Merge
50 μm

Supplement: S2 Fig — (A) WB confirmation of expression of HA-tagged TCV proteins. (B) Separate channel images of N. benthamiana leaf cells agro-infiltrated with a mixture of three constructs: Δp88ΔMP_sg2R + ΔMP_sg2G + p19. (PPTX) [file ppat.1006253.s002.pptx]

## Slide 1
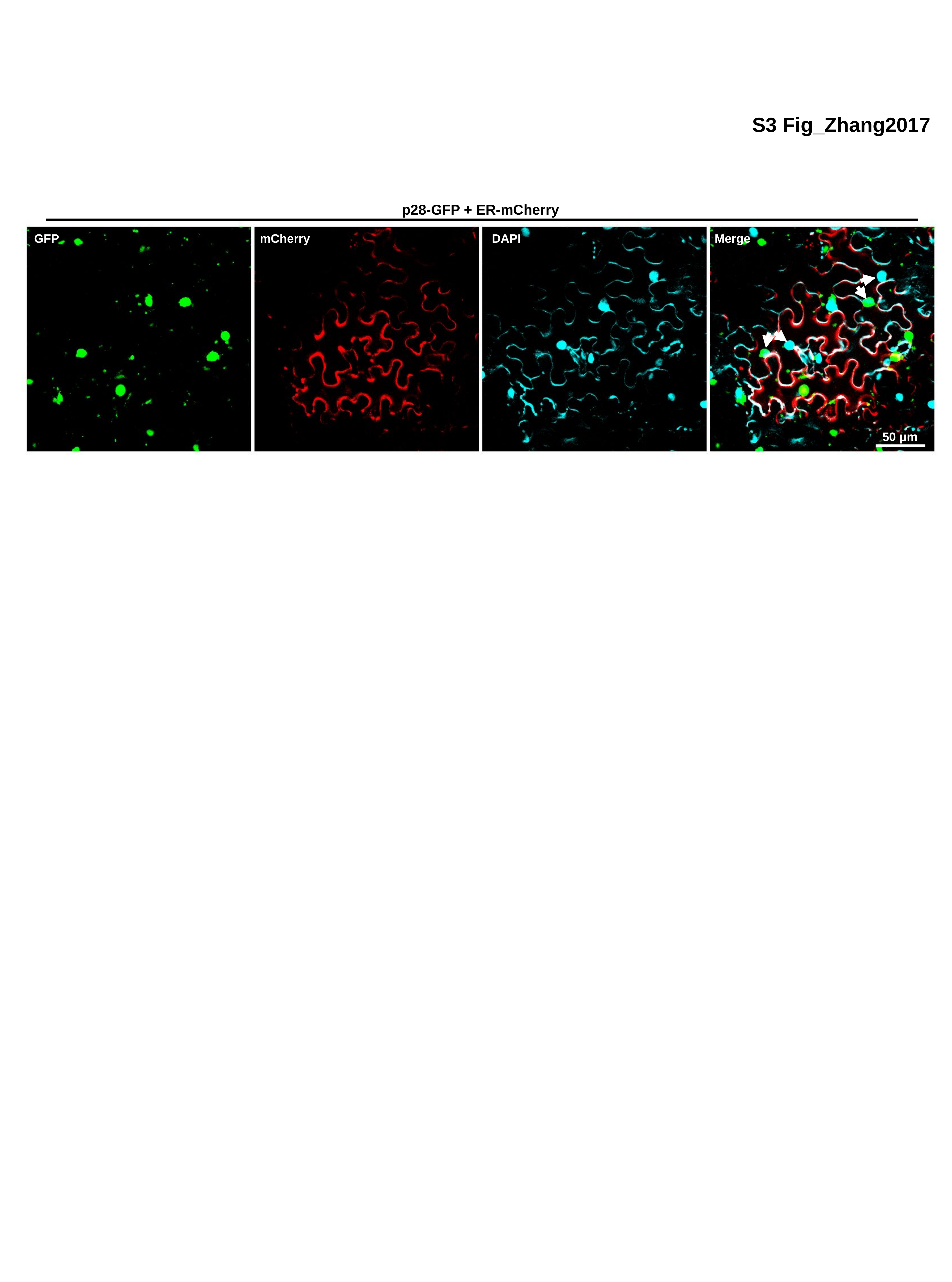

S3 Fig_Zhang2017
p28-GFP + ER-mCherry
Merge
GFP
mCherry
DAPI
50 μm

Supplement: S3 Fig — ER network was labelled with an ER-mCherry construct, and the cell nuclei (and cell wall) were stained with DAPI. Note the arrows in the Merge panel highlight the separation of p28-GFP foci from nuclei in two different cells. (PPTX) [file ppat.1006253.s003.pptx]
